# The Effect of UV-C Exposure on Larval Survival of the Dreissenid Quagga Mussel

**DOI:** 10.1371/journal.pone.0133039

**Published:** 2015-07-17

**Authors:** Alecia Stewart-Malone, Michael Misamore, Siri Wilmoth, Alejandro Reyes, Wai Hing Wong, Jackson Gross

**Affiliations:** 1 US Geological Survey, Northern Rocky Mountain Science Center, Bozeman, Montana, United States of America; 2 Texas Christian University, Fort Worth, Texas, United States of America; 3 Wilmoth Statistical Consulting, Bozeman, Montana, United States of America; 4 University of Nevada at Las Vegas, Las Vegas, Nevada, United States of America; University of California, Merced, UNITED STATES

## Abstract

The rapid spread of quagga mussels (*Dreissena rostriformis bugensis*) has lead to their invasion of Lake Mead, Nevada, the largest reservoir in North America and partially responsible for providing water to millions of people in the southwest. Current strategies for mitigating the growth and spread of quagga mussels primarily include physical and chemical means of removing adults within water treatment, delivery, and hydropower facilities. In the present study, germicidal ultraviolet light (UV-C) was used to target the larval stage of wild-caught quagga mussel. The lethal effect of UV-C was evaluated at four different doses, 0.0, 13.1, 26.2, and 79.6 mJ/cm^2^. Tested doses were determined based on results from preliminary trials. The results demonstrate that germicidal UV-C is effective in controlling the free-swimming life history stages of larval quagga mussels.

## Introduction

Dreissenid mussels are a non-native nuisance species in North America causing declines in local phytoplankton and zooplankton communities, marked changes in fish assemblages, and overall restructuring of entire lake ecosystems [1]. First sighted in 1989 in the Great Lakes, quagga mussels (*Dreissena rostriformis bugensis*) rapidly spread throughout the eastern United States [2,3,4]. In January 2007, quagga mussels were discovered in Lake Mead near Boulder City, Nevada [5,6]. Later that same month, quagga mussels were found downstream at Lakes Mohave and Havasu, confirming their infestation of the Lower Colorado River [6]. Together, these three reservoirs provide water for drinking, agricultural irrigation, flood control and hydroelectric power for approximately 25 million people across the southwest [7].

Biofouling is a consequence of mussel settlement and accumulated shell debris causing damage to boats and infrastructures, which require significant mitigation costs. The biofouling potential of Hoover and Davis Dam by quagga mussels warrants reassessment of the dams’ delivery of public services [8]. Hoover Dam, Boulder City, Nevada, supplies 15 million people with water and diverts water for over 1 million acres of irrigated land in California, Arizona and Mexico [9], and is located immediately downstream of Lake Mead. Davis Dam, located 67 miles downstream of Hoover, re-regulates Hoover Dam in order to meet downstream needs, including annual delivery to Mexico as stipulated by the 1944 Mexican Water Treaty [10]. Numerous structural components of dams and hydropower facilities are vulnerable to heavy macrofouling by quagga mussels including intake tower trash racks and cooling towers [8,11,12]. Efficiency of water processing facilities and water delivery can be substantially reduced due to mussel biofouling [11,12], thus effective methods to control biofouling need to be developed to preserve these vital services.

Current strategies for mitigating quagga mussel biofouling on dam structures include physical and chemical means of removing adults from the substrate [13,14]. While these methods can be effective at preventing adult colonization, they tend to be reactive in nature due to the fact that they target adults after settlement and after they have already caused a wide range of economic problems. Other methods of removing mussels include biofilter installation, drawing water below observed depth of settlement and decreasing dissolved oxygen (DO) concentrations in the flow water [15,11]. These techniques, in addition to physical and chemical removal, tend to be costly and labor intensive, and there is a lack of safe, effective and cost-efficient control methods [14,7]. Proactive strategies that target larval stages prior to settlement may allow for a wider range of dreissenid mussel biofouling control [16]. Successfully inhibiting the settlement and establishment of adults by inducing mortality at earlier life stages may prove to be an effective means of control.

Ultraviolet (UV) light technology has been used in the water treatment industry for years, having detrimental effects on aquatic organisms through DNA and protein damage [17]. Ultraviolet germicidal irradiation (UVGI) is a sterilization method using UV light at sufficiently short wavelengths to sterilize surfaces or fluid containing free swimming or pelagic microoganisms [6,18,19,20]. In controlled environments (e.g. hydropower facilities), where UV-C emission can be manipulated, exposure can be lethal to microorganisms through cellular damage [17,21,22,23]. If effective, UV-C radiation could prove to be a safe and effective, low cost, proactive method for mussel biofouling control in closed, piped systems (e.g. cooling water intakes). The purpose of this study was to establish a dose response relationship for UV-C radiation and quagga mussel larval survival 7 days post-exposure. It was expected that as UV-C dose increased, the magnitude of larval mortality would also increase, following a dose-response relationship.

## Materials and Methods

### 1.2.1 Ethics Statement

A National Park Service permit was obtained prior to beginning this work, granting permission to collect quagga mussels. Ethical approval was not required as this research did not involve endangered or protected species.

### 1.2.2 Study Animal

Quagga mussel larvae from all stages (trocophore, d-stage and umbonal veligers and pediveligers) were collected from Lake Mead at the Las Vegas Boat Harbor (36°1’48.70”N; 114°46’16.89”W) in Boulder basin. Larvae were collected approximately every third day from July through October 2012 using a conical vertical Wisconsin plankton net (sampling frequency determined based on lake larval concentrations). Each collection was emptied from the net into a container that held approximately 2 L of Lake Mead surface water and water quality was recorded (average temperature (°C), pH and DO (mg/L) were 25.6, 8.39 and 5.12, respectively). This sample container was then placed inside a 22.7-L bucket that contained approximately 3.8 L surface lake water to serve as a water bath to stabilize temperature during transport (9.7 km) to the Nevada Department of Wildlife’s (NDOW) Lake Mead Fish Hatchery, Boulder City, Nevada.

### 1.2.3 Pre-exposure

Upon arrival at the hatchery, water quality data were measured to ensure that conditions remained stable during transportation. This transported sample was concentrated (using a 64-μm mesh filter cup) to create a 200-mL sample. This concentrated sample was then quantified and subsequently diluted (using 20 μm filtered Lake Mead water (FLMW) obtained from inside hatchery) or further concentrated to achieve a 15–20 larvae/mL concentration. Average percent (%) UV-transmittance for this quantified, concentrated larvae sample, Lake Mead surface water and FLMW were 87.3, 88.0 and 88.6%, respectively. Transmittance was determined using a UVT meter (Real Tech Inc., Ontario, Canada). Only live larvae were included in quantification of the sample. Larvae were monitored for a maximum of 30 seconds for live or dead assessment: any larvae that were immobile (no cilia, internal organ, or body movement) were considered dead.

Ten mL of the concentrated larvae sample were transferred into clean 50-mL glass beakers (hereafter referred to as experimental beakers). Each experimental beaker represented an experimental unit, containing an estimated population of 150–200 mussel larvae. Ten 1-mL aliquots were sampled from different locations within the concentrated stock sample to ensure that the 10-mL subsample was an accurate representation of the whole quantified sample. Each experimental beaker was prepared one at a time to standardize and minimize the length of time that larvae spent in a concentrated 10-mL volume prior to UV-C exposure.

### 1.2.4 UV-C exposure

**Fig 1:**
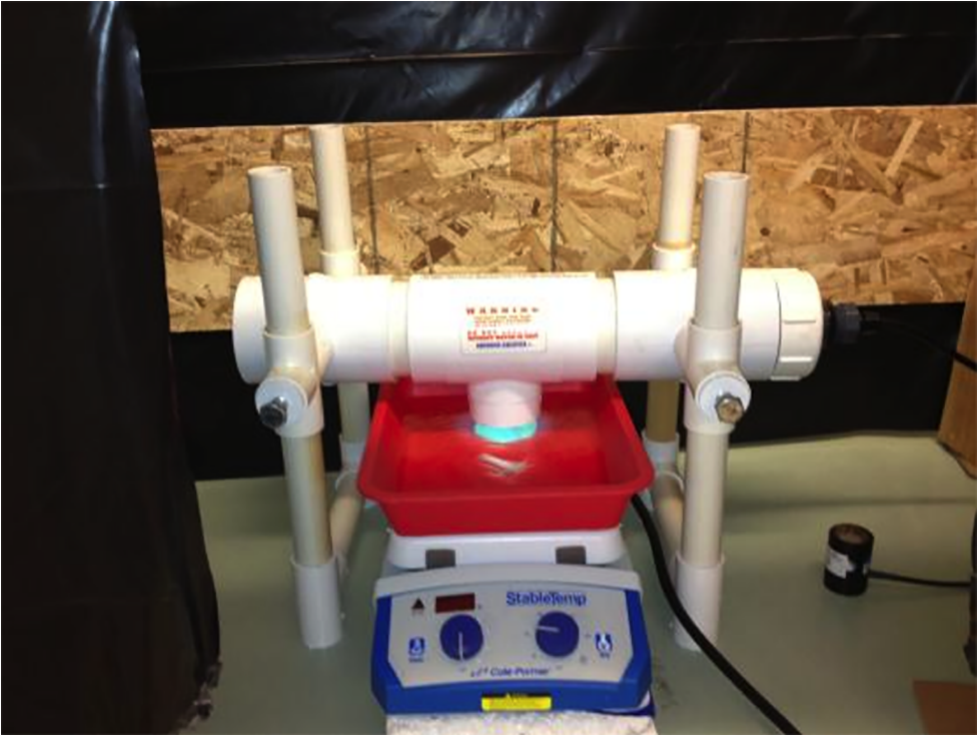
Laboratory setup. UV-C lamp set up as collimated beam. Data logging radiometer seen below lamp.

Larvae were exposed to UV-C from a tabletop low-pressure 50-watt amalgam lamp (Emperor Aquatics, Inc., Pottstown, PA, 19464) sheathed inside a hard, transparent quartz glass sleeve and set up as a collimated beam apparatus (see Fig 1 for experimental setup). Compared to standard low-pressure lamps, which typically use liquid mercury, amalgam lamps contain a chemical mixture bound to the lamp’s inner glass envelope wall, known as the fixed amalgam spot. This spot can create optimum mercury vapor pressure at 82.2°C, which allows for greater, more stable UV-C output over broad water temperature tolerances (1.6 to 48.9°C) (Emperor Aquatics Inc., 2010). With an overall UV-C output efficiency of 38–42%, the lamp’s 50-watt input generates 19 watts of emitted UV-C at 254 nm (Adrian Megay, personal communication, July 25, 2011). Radiation output was monitored and verified using a PMA2100 data logging radiometer (Solar Light Company, Inc., Glenside, PA 19038) before, during, and after all exposures. Hereafter, radiation output will be referred to as irradiance (μW/cm^2^).

Experimental beakers were exposed to UV-C one at a time, in a randomized order, 11.5 cm from the lamp. Length of exposure was calculated immediately before exposure of each beaker (due to small fluctuations in irradiance occurring throughout the experiment) using the following equation:

$$\text{Fluence}=\frac{\text{Irradiance}\times \text{Seconds exposure}}{1000}$$

Where fluence was the target dose (mJ/cm^2^) (total cumulative energy each sample of larvae received per time of exposure and hereafter referred to as dose), irradiance was the output measured by the radiometer just prior to the exposure of each beaker (μW/cm^2^), and seconds exposure was the number of seconds left underneath the lamp. Target doses were predetermined based on hypotheses derived from preliminary range finding experiments and covered a specific range of doses: 0, 13.1, 26.2, and 79.6 mJ/cm^2^ (exposure times for specific doses were approximately 0, 30, 60 and 170 seconds, respectively). Preliminary range finding experiments showed that for doses of 79.6, 133 and 260 mJ/cm^2^, 100% mortality could be achieved by 72, 72 and 48 hours post exposure (HPE), respectively.

A total of four experiments were conducted (see S1–S4 Datasheets for original data), each of which had 52 total beakers (for a total of 208 beakers – 64 control and 48 per exposure dose). Control beakers were placed next to the unit, where irradiance measured 0.00 μW/cm^2^. Both exposure and control beaker were placed inside 750-mL (1.5 cm depth) FLMW baths to prevent any changes in water temperature during exposure. The exposure beaker and bath were placed on a stir plate to enable continuous water circulation and to prevent temperature differences across locations in the bath. At the end of each exposure period, both beakers were immediately removed from their baths, diluted to 30 mL with fresh, ambient FLMW and placed into a 2-cm thick Styrofoam ring and floated inside a FLMW post-exposure bath to be analyzed at a later time. Water from both baths was replaced with fresh ambient FLMW before the next trial.

Pre- and post-exposure temperatures were compared to ensure that temperature remained constant. The range, mean and mean standard deviation pre-exposure temperatures in these beakers were 21.3–24.3°C, 22.8°C and 0.289, respectively. The range, mean and mean standard deviation post-exposure temperatures were 21.7–24.0°C, 22.8°C and 0.232, respectively.

Until time of analysis, all experimental beakers were kept in the FLMW post-exposure bath on a 12-h light cycle, located in an undisturbed room of the hatchery. Water temperatures were monitored daily to ensure it remained a constant 23°C.

### 1.2.5 Post-exposure & Data Collection

Mortality was assessed at post-exposure times of 36, 48, 60, 72, 96, 120, 144, and 168 hours (hereafter referred to as hours post-exposure, HPE) for control subsamples; 60, 72, 96, 120, 144, and 168 HPE for those receiving a dose of 13.1 mJ/cm^2^; 48, 60, 72, 96, 120, and 144 HPE for those receiving a dose of 26.2 mJ/cm^2^; and 36, 48, 60, 72, 96, and 120 HPE for those receiving a dose of 79.6 mJ/cm^2^. Different doses were assessed at particular HPE’s based on results of preliminary trials (e.g. mortality not seen in 13.1 mJ/cm^2^ samples until 60 HPE, etc.) To increase sampling and assessment efficiency, prior to each HPE assessment, each experimental beaker was concentrated from 30 mL to 10 mL with a Pasteur pipette and 64-μm mesh cup. Beaker contents were gently mixed and samples were taken from different locations within the beaker until ≥ 30 larvae had been counted–dead or alive. A larvae was considered dead if there was no visual cilia, internal organ, or body movement. Percent survival was calculated based on the mortality assay from the 30 sampled larvae. Every HPE for every dose had its own assigned experimental beaker so that each experimental beaker was sampled only once. During data collection, the treatment label on each beaker was covered with a piece of tape to reduce data collection bias. Beaker contents were immediately discarded after sampling.

Dissolved oxygen (mg/L) was measured in twenty-six randomly selected beakers. These 26 beakers were sampled on 5, 6 and 7 days post-exposure. Dissolved oxygen concentration (range, mean and mean standard deviation) for 5, 6 and 7 d post-exposure were 3.3–6.7, 4.5 and 1.400 mg/L; 2.9–5.0, 3.8, and 0.898 mg/L; and 3.2–4.7, 3.8 and 0.606 mg/L, respectively. Ammonia (mg/L) was measured in 6 randomly selected beakers (due to limited test kit supplies) and the range and mean ammonia for 5, 6 and 7 d post-exposure were 0.1–0.05 and 0.08 mg/L; 0.1–0.2 and 0.15 mg/L; and 0.2–0.2 and 0.2 mg/L, respectively. These data meet the requirement of water quality criteria for toxicity tests on dreissenid mussels and their veliger larvae [24].

### 1.2.6 Statistical Analysis

Data were analyzed from four replicated experiments (*n* = 4 experiments). For each experiment, all treatments (all possible dose and HPE combinations) had two replicate beakers (total of 208 beakers). Fisher’s Exact Tests were used to compare survival between the control group and each treatment group as well as between each treatment group. Logistic regression was used to estimate lethal dosages of UV-C for larval survival data. Logistic regression relates the probability of mortality (π) to dose and time variables through the logit link as follows: logit[π/(1 − π)] = β0 + β1 (log[dose]) + β2 (log[HPE]). In logistic regression, the estimated effect of a twenty-unit increase in dose is a [exp (β1*20)] multiplicative change on the odds of mortality after accounting for HPE and experiment [25]. Model selection was guided by plots of residuals, deviance goodness-of-fit tests, and drop-in-deviance *F*-tests.

A rich model, including interactions and higher order polynomials, failed a deviance goodness-of-fit (*P* < 0.0001, χ^2^ = 220.73, from a goodness-of-fit test of the deviance residuals with 123 *df*). Thus, the analysis compensated for that lack of fit by utilizing the quasilikelihood approach that inflates standard errors to account for extra variability contained in the data. To achieve linearity with the logit, data for the logistic regression analysis were excluded after 96 HPE. All terms in the final model were statistically significant at the α = 0.05 level with a pseudo-R^2^ of 0.661 [26].

## Results

**Fig 2:**
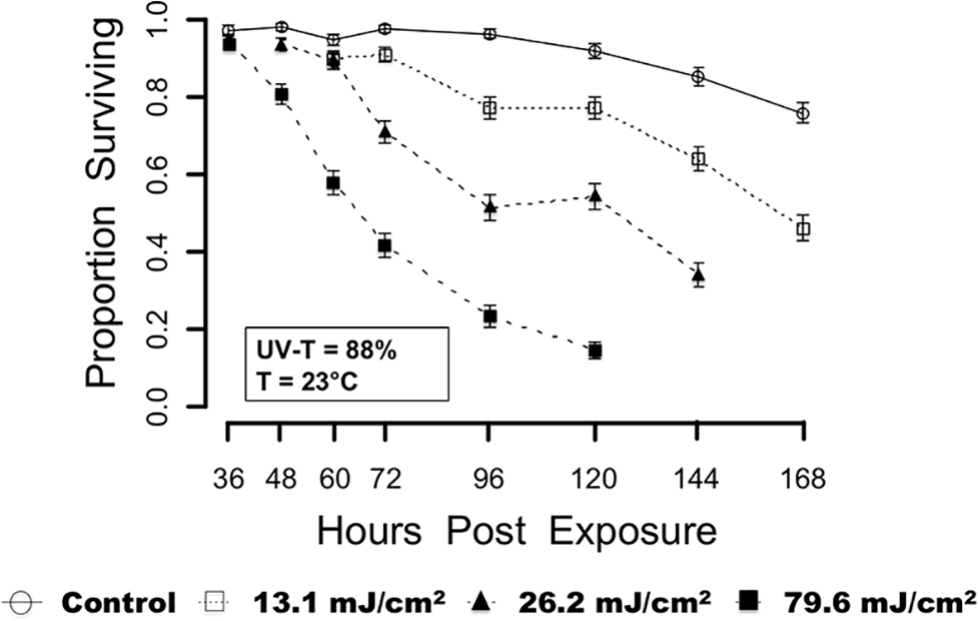
Mean proportion survival. Larval survival after exposure to one of four UV-C treatments. Mean ± SE bars shown.

For the lowest dose (13.1 mJ/cm^2^) at the first observation time (60 HPE), mortality was different than that of the control (*P* = 0.0429 from a Fisher’s Exact Test for equality between the control and treatment group) (Fig 2.). There were also significant differences between mortality in the 13.1 mJ/cm^2^ group and control at 72 HPE (*P* = 0.0014 from a Fisher’s Exact Test for equality between the control and treatment group). All other observation time points past 72 HPE exhibited significantly greater mortality than the control group (*P* < 0.0001 from Fisher’s Exact Tests). Larval mortality at 26.2 mJ/cm^2^ was significantly different from that of the control at every HPE. For the largest dose (79.6 mJ/cm^2^), mortality was not greater than that of the control at 36 HPE (*P* = 0.0897 from a Fisher’s Exact Test), but by 48 HPE mortality was significantly greater than controls (*P* < 0.0001 from a Fisher’s Exact Test) and for all subsequent HPE.

There was no evidence of a difference between the lowest dose (13.1 mJ/cm^2^) and 26.2 mJ/cm^2^ at 60 HPE (*P* = 0.4407 from Fisher’s Exact Tests). However, at 72 and 96 HPE mortality differences between 13.1 and 26.2 mJ/cm^2^ were statistically significant (*P* < 0.0001 (72 HPE) and *P* < 0.0001 (96 HPE) from two Fisher’s Exact Tests). There was overwhelming evidence to suggest differences in mortality caused by the highest dose (79.6 mJ/cm^2^) with all other treatment doses. At 60 and 72 HPE, there was significant mortality difference between the lowest and highest dose (*P* < 0.0001 (60 HPE) and *P* < 0.0001 (72 HPE), from two Fisher’s Exact Tests). At 48, 60 and 72 HPE, there was

significant mortality difference between the highest and middle (26.2 mJ/cm^2^) dose (*P* < 0.0001 (48 HPE), *P* < 0.0001 (60 HPE), and *P* < 0.0001 (72 HPE) from three Fisher’s Exact Tests).

After 96 HPE, statistical analyses revealed a significant decline in animal health within the controls (control survival at 120 HPE was significantly different from controls at 96 HPE (*P* = 0.0269), though the percentage values decreased from 95 to 92%), and increased mortality may to some extent have been the result of confounding factors in both control and experimental groups. For this reason and to meet model assumptions for the generalized linear model, data beyond 96 HPE were not used in predictive analyses.

Table 1 displays point estimates and 95% confidence intervals for exposure data up to 96 HPE required to achieve lethal dosages (LDs) for 25%, 50%, 75%, and 99% of the population of sampled larvae. Inferences are based on a quasibinomial logistic regression with interactions and polynomials from the entire data set up to 96 HPE.

**Table 1:** Lethal Dosage Estimates. Point estimates and their associated 95% confidence intervals for HPE required to achieve lethal dosages (LD) for larvae population.

Dose	LD25	LD50	LD75	LD99
13.1 mJ/cm^2^	101.6	145.1	207.2	644.0
	(91.4–115.1)	(126.7–174.2)	(172.9–268.1)	(452.0–1085.3)
26.2 mJ/cm^2^	78.8	108.1	148.2	404.5
	(73.3–85.0)	(99.2–120.4)	(131.5–174.0)	(313.1–579.7)
79.6 mJ/cm^2^	54.2	70.7	92.3	215.6
	(50.0–58.0)	(66.1–76.0)	(85.0–102.4)	(178.7–280.0)

## Discussion

### 1.4.1 UV-C and applicability

Since UV radiation, particularly UV-B and UV-C, have shorter wavelengths that can be absorbed by proteins and nucleic acids, it is capable of inducing lethal effects on aquatic organisms. In a controlled, laboratory setting (e.g. artificial lamps) UV-C can be utilized to cause lethal effects to microorganisms via cellular damage [17,18,19,20].

The present study illustrated the negative effects UV-C has on the survival of quagga mussel larvae in a laboratory setting. Since all water processed within a facility is drawn from intake pipes, mussel larvae could be exposed to artificially supplied UV radiation [27]. An important aspect of this application however, is the ability to achieve 100% mortality with significantly low exposure times and ideally with the least amount of energy. In the present study, the exposure times ranged from 30–170 seconds, but without 100% mortality. In Chalker-Scott et al. (1994) [27] and Wright et al. (1997) [28], though 100% mortality was achieved, it was done so with 1–20 minute exposures and with a significantly more powerful, less energy efficient lamp. The large differences in these variables across these three studies provide adequate room for improvement. More research still needs to be done in order to identify appropriate lamp specifications and exposure times that will elicit 100% mortality in an energy efficient manner.

### 1.4.2 UV comparison studies

Chalker-Scott et al. (1994) [27] and Wright et al. (1997) [28] are two of the more commonly cited dreissenid mussel UV exposure studies that provided the framework for the present study. Our results slightly overlap with their findings though large differences exist among all three studies, like fewer post-exposure mortality assessments (referred to as HPE in the present study) and greater UV lamp output and exposure times. In Chalker-Scott et al. (1994) [27], their lamp included many wavelengths of light (white light spectrum, plus UV-A and UV-B (< 280 nm)), allowing for the possibility of confounding effects from other UV wavelengths with the percentage of germicidal UV-C unknown. These broad-spectrum exposures were completed at a range of doses from 348–3067 mJ/cm^2^, which are orders of magnitude greater than those tested in the present study, and resulted in 100% mortality by 1 day post-exposure. In Wright et al. (1997) [28], the pure UV-C (254 nm) exposures were completed at 3 doses (702, 1404, and 2808 mJ/cm^2^), with percent mortality of 3, 15, and 34%, respectively at 24 HPE and 100% mortality at all doses by 168 HPE. In the present study, the highest dose (79.6 mJ/cm^2^) only caused 5.8% mortality at 36 HPE, but was near 80% at 96 HPE.

Differences in lamps across the three studies are an essential point of comparison. The low-pressure amalgam lamp used in the present study is primarily characterized by a low optimum vapor pressure of 1Pa. Other characteristics of this lamp include a UV-C output efficiency of near 40%, low electrical power input requirements of 40–500 W, low wall temperatures of 100°C, and a 16,000 hour lifetime [29]. Wright et al. (1997) [28] used a medium-pressure mercury lamp, which is primarily characterized by a higher optimum vapor pressure of 100 kPa, UV-C output efficiency of 5–15%, higher electrical power input requirements of 400–60 kW, extremely high lamp wall temperatures of 500–950°C, and a significantly lower lifetime of 5,000 hours [29]. Chalker-Scott et al. (1994) [27] used a high-pressure xenon arc lamp, which was 10 times more powerful (wattage) than the one in the present study and was characterized by higher operating temperatures as well. The lifetime of this type of lamp is approximately 5,000 hours [30]. In the presence of excess radiant heat from these high power lamps, dreissenid larvae can experience temperature shock, impacting their health [31,32], causing mortality as a result of high temperatures rather than exposure to UV. While a bed of crushed ice was used in Wright et al. (1997) [28] to control for any potential effects of radiant heat, no mention of a cool water bath was made in Chalker-Scott et al. (1994) [27]. Of the different UV lamps, Schalk et al. (2005) [29] states that in compact, economic systems, low-pressure amalgam lamps are the most efficient method of disinfection due to the large disparity between lamp types.

Interpretation on the effects of UV on mussels in the literature can have additional challenges due to differences in vocabulary, lack of comparable factors (e.g. lamp wattage, efficiency, power spectrum output variability), and differences in techniques for measuring UV, water quality and water temperature. In an attempt to most accurately draw comparisons, discrepancies between these studies need to be addressed further.

In some literature, lamp output as measured by a radiometer is referred to as irradiance, while in other literature the same units are referred to as fluence rate or light density. Also, the terms fluence and dose (both the total amount of light energy received at the target) are used interchangeably. Some literature report fluence or dose only as ‘minutes of exposure’, as in Wright et al. (1997) [28]. In some of these instances, important details such as actual lamp output (irradiance) and distance from the lamp are lacking, making it difficult to draw comparisons.

In addition to water temperature, other water quality parameters such as water transmissibility (UVT) were measured throughout the duration of the present study whereas it is not reported in the other studies. UVT, the total amount of UV light energy available for water treatment, is an important variable as it greatly impacts the bioavailability of light energy. For example, a 10% reduction in UVT from 100% to 90% can equate to greater than 36% reduction in bioavailable UV light [33].

In the present study, a 10-mL subsample of larvae with population density ranging from 15–20 larvae/mL was held in a 50-mL beaker. Chalker-Scott et al. (1994) [27] exposed only 10 larvae per trial in 10 mL of water held in a petri dish. Wright et al. (1997) [28] exposed 700 larvae in 10 mL of water held in a 30-mL beaker. Not only do the mussel densities vary across experiments, but the water depths and containment material vary as well, which could influence the effectiveness of the UV-C treatment.

Finally, while zebra and quagga mussel larvae are comparable in size and development, it is also possible that differences exist in the way they respond to UV light. Both previous studies used zebra mussels while the present study focused on quagga mussels. The response of one species may not be directly correlated to that of another as zebra and quagga mussels have been documented as being biologically different from each other [34,35]. Limited research exists evaluating DNA repair mechanism differences in dreissend mussels.

### 1.4.3 Conclusions

The use of germicidal UV shows promise as an effective tool to control quagga mussel larvae. The present study demonstrated that a dose as low as 26.2 mJ/cm^2^ can decrease survival of pre-settlement stage larvae by nearly 50% by 4 days after exposure. With a slighty stronger dose of 79.6 mJ/cm^2^, survival can be decreased by nearly 80% by 4 days after exposure. Overall with an increasing dose, survival of pre-settlement larvae decreased. This, to the authors’ knowledge, is the first dose response study on quagga mussels exposed to UV radiation.

Additional testing of the doses used in this experiment could prove helpful to further refine the dose response of quagga mussel larvae as well as extending the post-exposure assessment by a few additional days. For an assessment lasting longer than 120 hours, where control mortality exceeded 10%, a feeding and/or water change regiment would need to be added to the experimental design to help prevent increased mortality. Behavioral data of UV-C exposed quagga mussel larvae can be recorded alongside mortality data to allow further insight to the effects of irradiation. It is also of interest to assess how UV-C exposure influences settling ability. The process of substrate attachment and settling have high associated mortality costs; this factor could therefore be exploited by timing UV exposures during sensitive settling stages. Ecologically, impairments of pre-settlement mussel performance, including body mobility, food intake, and integrity of cellular functions, may be decisive for the survival of juvenile and adult life stages and consequently may affect the fitness dynamics of the whole population.

It is important to ultimately be able to expose specific age cohorts of mussels to UV-C in a laboratory setting so that its effects can be quantified for each larval stage of development. From this, it may be possible to identify particular stages that are more susceptible to UV radiation than others and develop a more effective management strategy by selecting the most conservative dose that would treat the most resilient life stage, and having an effect on all other stages.

## Supporting Information

S1 Datasheet Experiment One of Four. Collection data and exposure data from the first experiment.
(PDF)

S2 Datasheet Experiment Two of Four. Collection data and exposure data from the second experiment.
(PDF)

S3 Datasheet Experiment Three of Four. Collection data and exposure data from the third experiment.
(PDF)

S4 Datasheet Experiment Four of Four. Collection data and exposure data from the fourth experiment.
(PDF)

S1 Table Summary Figures. Percent survival results from each individual experiment (one through four).
(PDF)
