# Supplementary material for: The Effect of UV-C Exposure on Larval Survival of the Dreissenid Quagga Mussel
Source: PLoS One. 2015 Jul 17;10(7):e0133039. doi: 10.1371/journal.pone.0133039 (PMC4505903; doi:10.1371/journal.pone.0133039)

7:00 am tow (13)			Plankton tow sample	
LMW UV-T	88.8		Pre transport t	26
FLMW UV-T	91.2		Pre transport pH	8.47
Quant sample UV-T	89.7		temp	25.6
			DO	6.1
Quantification	16.8Vel/ml	at 1000ml	pH	8.45

**Beaker ID**	**Pre-Exp bath temp (°C)**	**Post-Exp bath temp (°C)**	**ΔT**	**Target Fluence**	**Irradiance**
36-0A	24.1	24	0.1	0.0	0
36-0B	24	23.6	0.4	0.0	0
36-79.6A	24	23.9	0.1	79.6	495
36-79.6B	23.9	23.6	0.3	79.6	496
48-0A	23.6	23.4	0.2	0.0	0
48-0B	23.4	23.3	0.1	0.0	0
48-26.2A	23.6	23.4	0.2	26.2	499
48-26.2B	23.4	23.4	0.0	26.2	499
48-79.6A	23.4	23	0.4	79.6	498
48-79.6B	23	22.9	0.1	79.6	499
60-0A	22.7	22.4	0.3	0.0	0
60-0B	22.4	22.3	0.1	0.0	0
60-13.1A	22.4	22.6	-0.2	13.1	498
60-13.1B	22.5	22.5	0.0	13.1	496
60-26.2A	22.5	22.4	0.1	26.2	494
60-26.2B	22.4	22.4	0.0	26.2	494
60-79.6A	22.4	22.3	0.1	79.6	492
60-79.6B	22.3	22.2	0.1	79.6	494
72-0A	22.2	22.3	-0.1	0.0	0
72-0B	22.3	22.2	0.1	0.0	0
72-13.1A	22.3	22.4	-0.1	13.1	496
72-13.1B	22.3	22.2	0.1	13.1	498
72-26.2A	22.2	22.1	0.1	26.2	501
72-26.2B	22.1	22.1	0.0	26.2	502
72-79.6A	22.1	22	0.1	79.6	502
72-79.6B	22	22	0.0	79.6	500
96-0A	24.3	24	0.3	0.0	0
96-0B	24	24	0.0	0.0	0
96-13.1A	24	23.9	0.1	13.1	488
96-13.1B	23.9	23.7	0.2	13.1	496
96-26.2A	23.7	23.7	0.0	26.2	494
96-26.2B	23.7	23.6	0.1	26.2	490
96-79.6A	23.6	23.4	0.2	79.6	482
96-79.6B	23.4	23.3	0.1	79.6	487

120-0A	23.3	23.4	-0.1	0	0
120-0B	23.4	23.1	0.3	0	0
120-13.1A	23.1	23	0.1	13.1	489
120-13.1B	23	23	0.0	13.1	480
120-26.2A	23	23.1	-0.1	26.2	477
120-26.2B	23.1	22.9	0.2	26.2	482
120-79.6A	22.9	22.9	0.0	79.6	484
120-79.6B	22.9	22.8	0.1	79.6	484
144-0A	22.8	22.9	-0.1	0	0
144-0B	22.9	22.9	0.0	0	0
144-13.1A	22.6	22.6	0.0	13.1	487
144-13.1B	22.6	22.6	0.0	13.1	488
144-26.2A	22.5	22.5	0.0	26.2	490
144-26.2B	22.5	22.3	0.2	26.2	490
168-0A	22.3	22.2	0.1	0	0
168-0B	22.2	22.1	0.1	0	0
168-13.1A	22.5	22.3	0.2	13.1	489
168-13.1B	22.3	22.2	0.1	13.1	486

Lake conditions

		Began Exposure	926
pH	8.59	End exposure	1145
temp	26.8		

Ammonium 0.175

Seconds exposure	# mLs sampled	Counted # alive	Total # counted	Proportion survival
0	6	28	30	0.93
0	2	30	30	1.00
160.808081	2	30	30	1.00
160.483871	2	30	30	1.00
0	2	30	30	1.00
0	2	27	30	0.90
52.50501	2	30	30	1.00
52.50501	2	26	30	0.87
159.839357	2	27	30	0.90
159.519038	2	27	30	0.90
0	4	29	30	0.97
0	2	26	30	0.87
26.3052209	2	22	30	0.73
26.4112903	2	25	30	0.83
53.0364372	4	27	30	0.90
53.0364372	4	24	30	0.80
161.788618	2	18	30	0.60
161.133603	2	20	30	0.67
0	2	28	30	0.93
0	2	29	30	0.97
26.4112903	2	25	30	0.83
26.3052209	2	26	30	0.87
52.2954092	2	21	30	0.70
52.1912351	2	18	30	0.60
158.565737	2	19	30	0.63
159.2	2	11	30	0.37
0	2	28	30	0.93
0	4	30	30	1.00
26.8442623	2	24	30	0.80
26.4112903	2	14	30	0.47
53.0364372	4	7	30	0.23
53.4693878	2	16	30	0.53
165.145228	2	8	30	0.27
163.449692	4	6	30	0.20

Fluence mJ/cm2
0.0
13.1
26.2
79.6

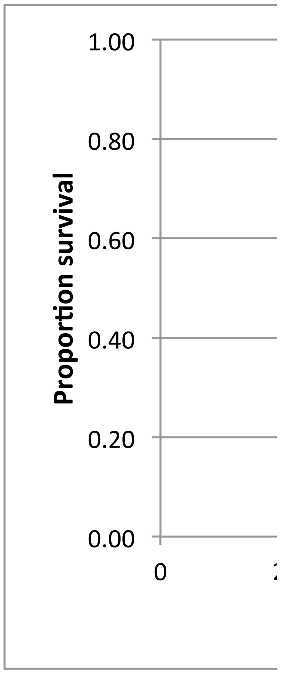

0	4	24	30	0.80
0	2	29	30	0.97
26.7893661	2	27	30	0.90
27.2916667	2	23	30	0.77
54.9266247	2	16	30	0.53
54.3568465	4	12	30	0.40
164.46281	4	11	30	0.37
164.46281	4	5	30	0.17
0	2	28	30	0.93
0	2	15	30	0.50
26.899384	8	19	30	0.63
26.8442623	2	22	30	0.73
53.4693878	4	8	30	0.27
53.4693878	4	6	30	0.20
0	4	19	30	0.63
0	4	22	30	0.73
26.7893661	2	8	30	0.27
26.9547325	2	26	30	0.87

36	48	60	72	96	120	144	168
0.97	0.95	0.92	0.95	0.97	0.88	0.72	0.68
		0.78	0.85	0.63	0.83	0.68	0.57
	0.93	0.85	0.65	0.38	0.47	0.23	
1.00	0.90	0.63	0.50	0.23	0.27		

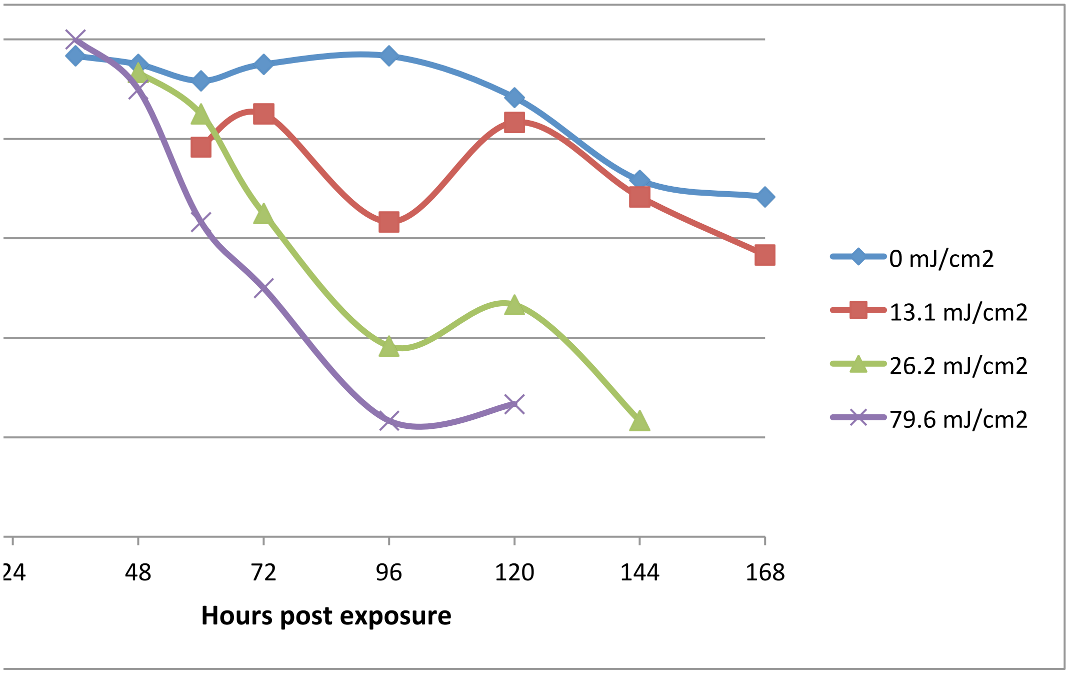

Supplement: S1 Datasheet — Collection data and exposure data from the first experiment. (PDF) [file pone.0133039.s001.pdf]
