# Supplementary material for: The Effect of UV-C Exposure on Larval Survival of the Dreissenid Quagga Mussel
Source: PLoS One. 2015 Jul 17;10(7):e0133039. doi: 10.1371/journal.pone.0133039 (PMC4505903; doi:10.1371/journal.pone.0133039)

5:45pm tow (13)			Plankton tow sample		
LMW UV-T	88.5		Pre transport t	27.9	
FLMW UV-T	86.2		Pre transport pH	8.58	
Quant sample UV-T	85.5		Pre transport DO	3.8	
Ammonia	0.2		Post temp	27.4	
Quantification	16.8 vel/mL	600mLs	Post DO	4.5	
			Post pH	8.46	

**Beaker ID**	**Pre-Exp bath temp (°C)**	**Post-Exp bath temp (°C)**	**ΔT**	**Target Fluence**	**Irradiance**
36-0A	22.4	22.3	0.1	0.0	0
36-0B	22.3	22.2	0.1	0.0	0
36-79.6A	22.3	22.4	-0.1	79.6	493
36-79.6B	22.4	22.5	-0.1	79.6	494
48-0A	22.2	22.2	0.0	0.0	0
48-0B	22.2	22.1	0.1	0.0	0
48-26.2A	22.5	22.4	0.1	26.2	495
48-26.2B	22.4	22.5	-0.1	26.2	498
48-79.6A	22.5	22.5	0.0	79.6	497
48-79.6B	22.5	22.5	0.0	79.6	497
60-0A	22.2	22.3	-0.1	0.0	0
60-0B	22.3	22.3	0.0	0.0	0
60-13.1A	22.1	22.2	-0.1	13.1	493
60-13.1B	22.5	22.6	-0.1	13.1	498
60-26.2A	22.2	22.2	0.0	26.2	500
60-26.2B	22.1	22.2	-0.1	26.2	500
60-79.6A	22.4	22.5	-0.1	79.6	503
60-79.6B	22.5	22.6	-0.1	79.6	503
72-0A	22.4	22.2	0.2	0.0	0
72-0B	22.3	22.4	-0.1	0.0	0
72-13.1A	22.3	22.2	0.1	13.1	502
72-13.1B	22.2	22.3	-0.1	13.1	502
72-26.2A	22.3	22.3	0.0	26.2	502
72-26.2B	22.3	22.3	0.0	26.2	498
72-79.6A	22.4	22.5	-0.1	79.6	495
72-79.6B	22.5	22.5	0.0	79.6	492
96-0A	22.6	22.5	0.1	0.0	0
96-0B	22.5	22.6	-0.1	0.0	0
96-13.1A	22.4	22.4	0.0	13.1	492
96-13.1B	22.4	22.5	-0.1	13.1	493
96-26.2A	22.5	22.5	0.0	26.2	494
96-26.2B	22.5	22.5	0.0	26.2	489
96-79.6A	22.5	22.5	0.0	79.6	494
96-79.6B	22.5	22.7	-0.2	79.6	485

120-0A	22.8	22.8	0.0	0	0
120-0B	22.8	22.7	0.1	0	0
120-13.1A	22.6	22.5	0.1	13.1	490
120-13.1B	22.5	22.5	0.0	13.1	492
120-26.2A	22.5	22.5	0.0	26.2	489
120-26.2B	22.5	22.5	0.0	26.2	490
120-79.6A	22.7	22.8	-0.1	79.6	484
120-79.6B	22.8	22.9	-0.1	79.6	487
144-0A	22.8	22.8	0.0	0	0
144-0B	22.8	22.8	0.0	0	0
144-13.1A	22.7	22.7	0.0	13.1	485
144-13.1B	22.7	22.7	0.0	13.1	488
144-26.2A	22.8	22.8	0.0	26.2	480
144-26.2B	22.8	22.9	-0.1	26.2	471
168-0A	22.8	22.7	0.1	0	0
168-0B	22.7	22.7	0.0	0	0
168-13.1A	22.9	22.8	0.1	13.1	474
168-13.1B	22.8	22.8	0.0	13.1	479

Lake conditions
calm, some clouds, 95 air temp

pH	8.41
temp	27.8
DO	6.53

Began Exposure	7:40pm
End exposure	9:40pm

Ammonia 0.20 mg/L

Seconds exposure	# mLs sampled	Counted # alive	Total # counted	Proportion survival
0	2	30	30	1.00
0	2	29	30	0.97
161.460446	2	26	30	0.87
161.133603	4	28	30	0.93
0	6	29	30	0.97
0	2	29	30	0.97
52.9292929	4	26	30	0.87
52.6104418	2	26	30	0.87
160.160966	6	17	30	0.57
160.160966	4	20	30	0.67
0	4	30	30	1.00
0	6	28	30	0.93
26.5720081	4	29	30	0.97
26.3052209	4	29	30	0.97
52.4	4	27	30	0.90
52.4	4	26	30	0.87
158.250497	4	15	30	0.50
158.250497	6	11	30	0.37
0	2	29	30	0.97
0	4	30	30	1.00
26.0956175	4	29	30	0.97
26.0956175	4	27	30	0.90
52.1912351	4	20	30	0.67
52.6104418	4	24	30	0.80
160.808081	4	7	30	0.23
161.788618	6	11	30	0.37
0	4	26	30	0.87
0	6	29	30	0.97
26.6260163	4	24	30	0.80
26.5720081	4	27	30	0.90
53.0364372	2	16	30	0.53
53.5787321	4	17	30	0.57
161.133603	4	2	30	0.07
164.123711	4	3	30	0.10

Fluence mJ/cm2
0.0
13.1
26.2
79.6

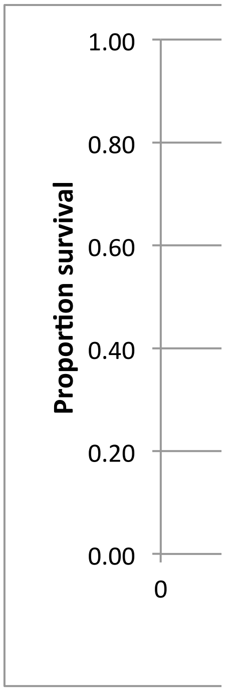

0	4	25	30	0.83
0	4	28	30	0.93
26.7346939	2	23	30	0.77
26.6260163	2	10	30	0.33
53.5787321	2	20	30	0.67
53.4693878	4	16	30	0.53
164.46281	4	3	30	0.10
163.449692	2	1	30	0.03
0	2	24	30	0.80
0	4	23	30	0.77
27.0103093	4	23	30	0.77
26.8442623	4	10	30	0.33
54.5833333	4	5	30	0.17
55.626327	4	12	30	0.40
0	4	19	30	0.63
0	4	19	30	0.63
27.6371308	4	18	30	0.60
27.348643	4	8	30	0.27

36	48	60	72	96	120	144	168
0.98	0.97	0.97	0.98	0.92	0.88	0.78	0.63
		0.97	0.93	0.85	0.55	0.55	0.43
	0.87	0.88	0.73	0.55	0.60	0.28	
0.90	0.62	0.43	0.30	0.08	0.07		

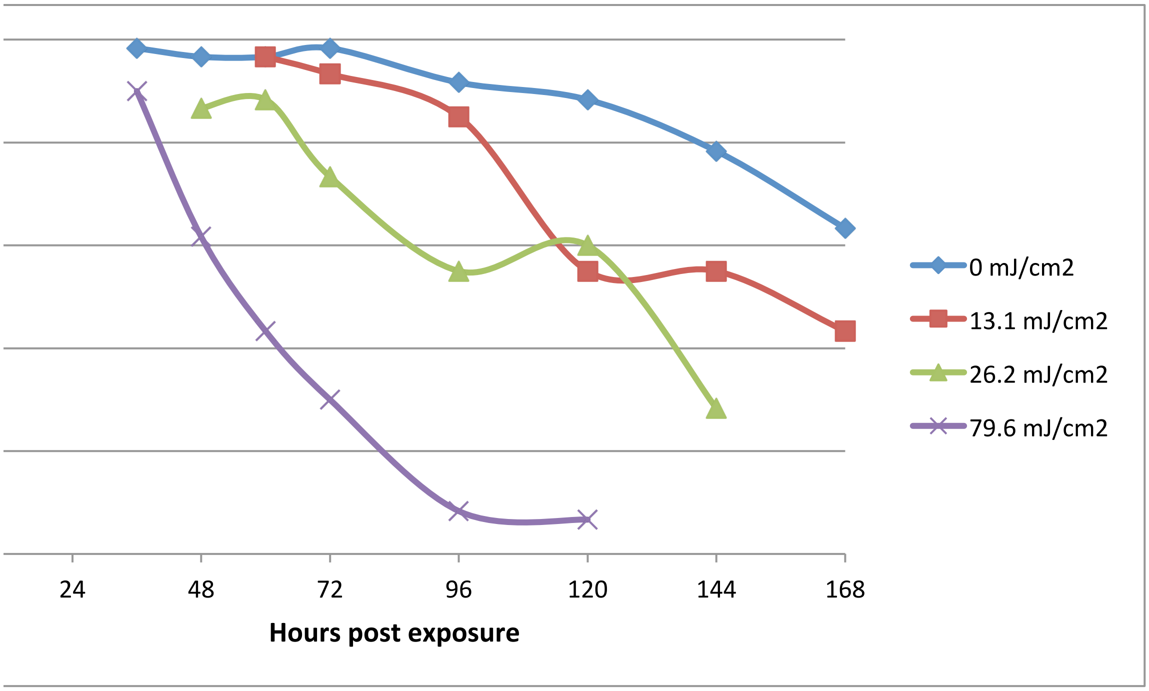

Supplement: S2 Datasheet — Collection data and exposure data from the second experiment. (PDF) [file pone.0133039.s002.pdf]
