# Supplementary material for: The Effect of UV-C Exposure on Larval Survival of the Dreissenid Quagga Mussel
Source: PLoS One. 2015 Jul 17;10(7):e0133039. doi: 10.1371/journal.pone.0133039 (PMC4505903; doi:10.1371/journal.pone.0133039)

5:45pm tow (13)				Plankton tow sample	
LMW UV-T	86.4			Pre transport t	24.7
FLMW UV-T	87.9			Pre transport pH	8.21
Quant sample UV-T	85.3			Pre transport DO	3.76
Ammonia	n/a			Post temp	24
Quantification	17.2vel/mL	800mL		Post DO	3.68
				Post pH	8.26

Beaker ID	Pre-Exp bath temp (°C)	Post-Exp bath temp (°C)	ΔT	Target Fluence	Irradiance
36-0A	21.3	21.7	-0.4	0.0	0
36-0B	21.7	21.7	0.0	0.0	0
36-79.6A	21.4	21.8	-0.4	79.6	474
36-79.6B	21.8	22	-0.2	79.6	476
48-0A	22.1	22.2	-0.1	0.0	0
48-0B	22.2	22.2	0.0	0.0	0
48-26.2A	21.8	22.1	-0.3	26.2	480
48-26.2B	22.1	22.1	0.0	26.2	480
48-79.6A	22	22.1	-0.1	79.6	477
48-79.6B	22.1	22.2	-0.1	79.6	480
60-0A	22.4	22.4	0.0	0.0	0
60-0B	22.4	22.5	-0.1	0.0	0
60-13.1A	22.2	22.3	-0.1	13.1	478
60-13.1B	22.2	22.3	-0.1	13.1	478
60-26.2A	22.2	22.3	-0.1	26.2	475
60-26.2B	22.3	22.4	-0.1	26.2	475
60-79.6A	22.3	22.5	-0.2	79.6	474
60-79.6B	22.5	22.6	-0.1	79.6	476
72-0A	22.6	22.7	-0.1	0.0	0
72-0B	22.7	22.7	0.0	0.0	0
72-13.1A	22.5	22.6	-0.1	13.1	474
72-13.1B	22.4	22.7	-0.3	13.1	474
72-26.2A	22.6	22.6	0.0	26.2	471
72-26.2B	22.7	22.7	0.0	26.2	471
72-79.6A	22.7	22.8	-0.1	79.6	472
72-79.6B	22.8	22.8	0.0	79.6	474
96-0A	22.8	22.9	-0.1	0.0	0
96-0B	22.9	22.8	0.1	0.0	0
96-13.1A	22.7	22.8	-0.1	13.1	473
96-13.1B	22.8	22.9	-0.1	13.1	473
96-26.2A	22.8	22.8	0.0	26.2	475
96-26.2B	22.9	23	-0.1	26.2	475
96-79.6A	23	23.1	-0.1	79.6	472
96-79.6B	23.1	23.2	-0.1	79.6	472

120-0A	23	22.9	0.1	0	0
120-0B	22.9	23	-0.1	0	0
120-13.1A	22.8	23	-0.2	13.1	474
120-13.1B	23.2	23.2	0.0	13.1	474
120-26.2A	23	23	0.0	26.2	473
120-26.2B	23.2	23.2	0.0	26.2	473
120-79.6A	23.2	23.2	0.0	79.6	467
120-79.6B	23.2	23.3	-0.1	79.6	468
144-0A	23.1	23	0.1	0	0
144-0B	23	23	0.0	0	0
144-13.1A	23	23.1	-0.1	13.1	456
144-13.1B	23.3	23.3	0.0	13.1	456
144-26.2A	23.3	23.2	0.1	26.2	467
144-26.2B	23.2	23.3	-0.1	26.2	467
168-0A	23	23.1	-0.1	0	0
168-0B	23.1	23.1	0.0	0	0
168-13.1A	23.3	23.3	0.0	13.1	458
168-13.1B	23.3	23.3	0.0	13.1	457

Lake conditions
cloudy, windy, slight rain
70 degrees F

pH	8.24
temp	25.6
DO	6.18

Began Exposure	8:00pm
End exposure	9:30pm

Seconds exposure	# mLs sampled	Counted # alive	Total # counted	Proportion survival
0	2	28	30	0.93
0	4	28	30	0.93
167.932489	2	28	30	0.93
167.226891	4	25	30	0.83
0	2	30	30	1.00
0	2	30	30	1.00
54.5833333	4	29	30	0.97
54.5833333	2	29	30	0.97
166.87631	4	25	30	0.83
165.833333	4	24	30	0.80
0	4	30	30	1.00
0	2	30	30	1.00
27.4058577	4	27	30	0.90
27.4058577	4	29	30	0.97
55.1578947	4	28	30	0.93
55.1578947	2	28	30	0.93
167.932489	2	16	30	0.53
167.226891	4	22	30	0.73
0	4	30	30	1.00
0	2	29	30	0.97
27.6371308	2	28	30	0.93
27.6371308	4	29	30	0.97
55.626327	2	22	30	0.73
55.626327	2	26	30	0.87
168.644068	4	15	30	0.50
167.932489	4	13	30	0.43
0	4	28	30	0.93
0	2	30	30	1.00
27.6955603	2	27	30	0.90
27.6955603	2	24	30	0.80
55.1578947	2	18	30	0.60
55.1578947	2	21	30	0.70
168.644068	2	13	30	0.43
168.644068	4	8	30	0.27

Fluence mJ/cm2
0.0
13.1
26.2
79.6

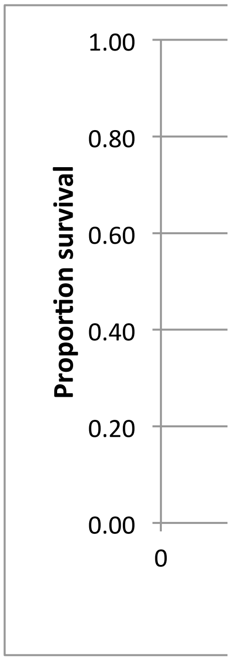

0	2	29	30	0.97
0	2	29	30	0.97
27.6371308	2	28	30	0.93
27.6371308	2	28	30	0.93
55.3911205	4	15	30	0.50
55.3911205	2	20	30	0.67
170.449679	2	2	30	0.07
170.08547	4	8	30	0.27
0	2	28	30	0.93
0	2	28	30	0.93
28.7280702	2	27	30	0.90
28.7280702	2	25	30	0.83
56.1027837	2	11	30	0.37
56.1027837	2	20	30	0.67
0	2	24	30	0.80
0	2	26	30	0.87
28.6026201	2	19	30	0.63
28.6652079	2	12	30	0.40

36	48	60	72	96	120	144	168
0.93	1.00	1.00	0.98	0.97	0.97	0.93	0.83
		0.93	0.95	0.85	0.93	0.87	0.52
	0.97	0.93	0.80	0.65	0.58	0.52	
0.88	0.82	0.63	0.47	0.35	0.17		

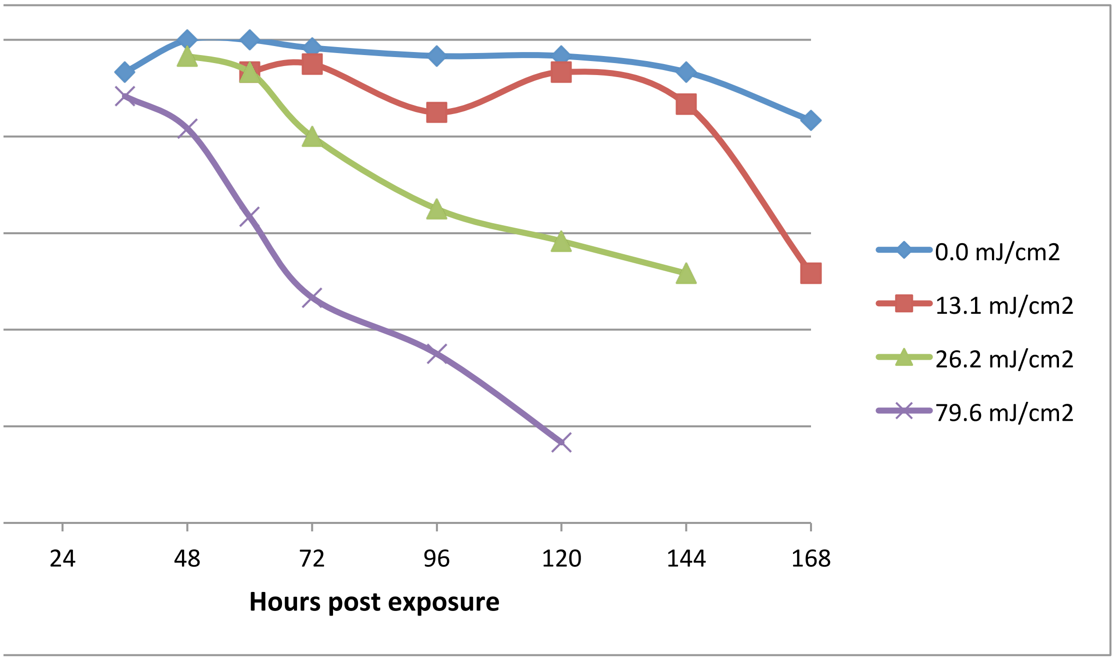

Supplement: S3 Datasheet — Collection data and exposure data from the third experiment. (PDF) [file pone.0133039.s003.pdf]
