# Supplementary material for: The Effect of UV-C Exposure on Larval Survival of the Dreissenid Quagga Mussel
Source: PLoS One. 2015 Jul 17;10(7):e0133039. doi: 10.1371/journal.pone.0133039 (PMC4505903; doi:10.1371/journal.pone.0133039)

7:15am tow (15)			Plankton tow sample	
LMW UV-T	88.3		Pre transport t	23.8
FLMW UV-T	89.1		Pre transport pH	8.36
Quant sample UV-T	89.3		Pre transport DO	7.81
			Post temp	23.5
Quantification	16.8 vel/mL	1L	Post DO	8.12
			Post pH	8.2

**Beaker ID**	**Pre-Exp bath temp (°C)**	**Post-Exp bath temp (°C)**	**ΔT**	**Target Fluence**	**Irradiance**
36-0A	23.6	23.3	0.3	0.0	0
36-0B	23.3	23	0.3	0.0	0
36-79.6A	23.6	23.2	0.4	79.6	462
36-79.6B	23.2	23.2	0.0	79.6	468
48-0A	23	23	0.0	0.0	0
48-0B	23	22.9	0.1	0.0	0
48-26.2A	23.2	23.1	0.1	26.2	470
48-26.2B	23.1	23.1	0.0	26.2	471
48-79.6A	23.1	23.1	0.0	79.6	474
48-79.6B	23.1	23	0.1	79.6	476
60-0A	23.2	22.9	0.3	0.0	0
60-0B	22.9	22.7	0.2	0.0	0
60-13.1A	23	22.9	0.1	13.1	477
60-13.1B	22.9	22.7	0.2	13.1	476
60-26.2A	22.7	22.8	-0.1	26.2	482
60-26.2B	22.8	22.8	0.0	26.2	480
60-79.6A	22.8	22.7	0.1	79.6	480
60-79.6B	22.7	23	-0.3	79.6	480
72-0A	23.1	23	0.1	0.0	0
72-0B	23	23	0.0	0.0	0
72-13.1A	23	23	0.0	13.1	479
72-13.1B	23	23.1	-0.1	13.1	482
72-26.2A	23.1	23.1	0.0	26.2	477
72-26.2B	23.1	23	0.1	26.2	477
72-79.6A	23	22.8	0.2	79.6	470
72-79.6B	22.8	22.9	-0.1	79.6	474
96-0A	23	23	0.0	0.0	0
96-0B	23	22.9	0.1	0.0	0
96-13.1A	22.9	22.7	0.2	13.1	481
96-13.1B	22.7	22.6	0.1	13.1	480
96-26.2A	23.6	23.5	0.1	26.2	483
96-26.2B	23.5	23.3	0.2	26.2	479
96-79.6A	23.3	23.3	0.0	79.6	479
96-79.6B	23.3	23.5	-0.2	79.6	478

120-0A	23.6	23.5	0.1	0	0
120-0B	23.5	23.5	0.0	0	0
120-13.1A	23.5	23.4	0.1	13.1	489
120-13.1B	23.4	23.4	0.0	13.1	484
120-26.2A	23.4	23.3	0.1	26.2	477
120-26.2B	23.3	23.5	-0.2	26.2	481
120-79.6A	23.5	23.4	0.1	79.6	480
120-79.6B	23.4	23.4	0.0	79.6	479
144-0A	23.4	23.4	0.0	0	0
144-0B	23.4	23.2	0.2	0	0
144-13.1A	23.2	23	0.2	13.1	470
144-13.1B	23	22.9	0.1	13.1	471
144-26.2A	22.9	22.9	0.0	26.2	472
144-26.2B	22.8	22.7	0.1	26.2	470
168-0A	22.7	22.7	0.0	0	0
168-0B	22.7	22.8	-0.1	0	0
168-13.1A	22.8	22.8	0.0	13.1	469
168-13.1B	22.8	22.7	0.1	13.1	474

Lake conditions
Calm, little wind, started to pick up at end

pH	8.2
temp	25.1
DO	7.67

Began Exposure	9:20
End exposure	11:40

**Seconds exposure**	**# mLs sampled**	**Counted # alive**	**Total # counted**	**Proportion survival**
0	2	30	30	1.00
0	2	30	30	1.00
172.294372	2	29	30	0.97
170.08547	4	30	30	1.00
0	2	30	30	1.00
0	2	30	30	1.00
55.7446809	2	30	30	1.00
55.626327	2	29	30	0.97
167.932489	2	27	30	0.90
167.226891	2	26	30	0.87
0	4	27	30	0.90
0	4	27	30	0.90
27.4633124	2	26	30	0.87
27.5210084	2	29	30	0.97
54.3568465	2	27	30	0.90
54.5833333	4	27	30	0.90
165.833333	2	15	30	0.50
165.833333	4	22	30	0.73
0	2	30	30	1.00
0	2	29	30	0.97
27.348643	2	27	30	0.90
27.1784232	2	27	30	0.90
54.9266247	2	22	30	0.73
54.9266247	2	17	30	0.57
169.361702	2	14	30	0.47
167.932489	2	10	30	0.33
0	2	30	30	1.00
0	2	30	30	1.00
27.2349272	4	24	30	0.80
27.2916667	2	21	30	0.70
54.2443064	4	15	30	0.50
54.697286	2	13	30	0.43
166.179541	2	7	30	0.23
166.527197	4	9	30	0.30

Fluence mJ/cm2
0.0
13.1
26.2
79.6

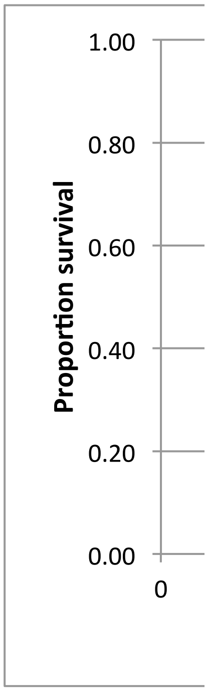

0	2	29	30	0.97
0	2	27	30	0.90
26.7893661	2	24	30	0.80
27.0661157	4	22	30	0.73
54.9266247	2	17	30	0.57
54.4698545	2	14	30	0.47
165.833333	4	2	30	0.07
166.179541	2	3	30	0.10
0	2	29	30	0.97
0	2	29	30	0.97
27.8723404	4	19	30	0.63
27.8131635	4	8	30	0.27
55.5084746	2	12	30	0.40
55.7446809	4	8	30	0.27
0	4	25	30	0.83
0	4	28	30	0.93
27.9317697	4	10	30	0.33
27.6371308	4	10	30	0.33

36	48	60	72	96	120	144	168
1.00	1.00	0.90	0.98	1.00	0.93	0.97	0.88
		0.92	0.90	0.75	0.77	0.45	0.33
	0.98	0.90	0.65	0.47	0.52	0.33	
0.98	0.88	0.62	0.40	0.27	0.08		

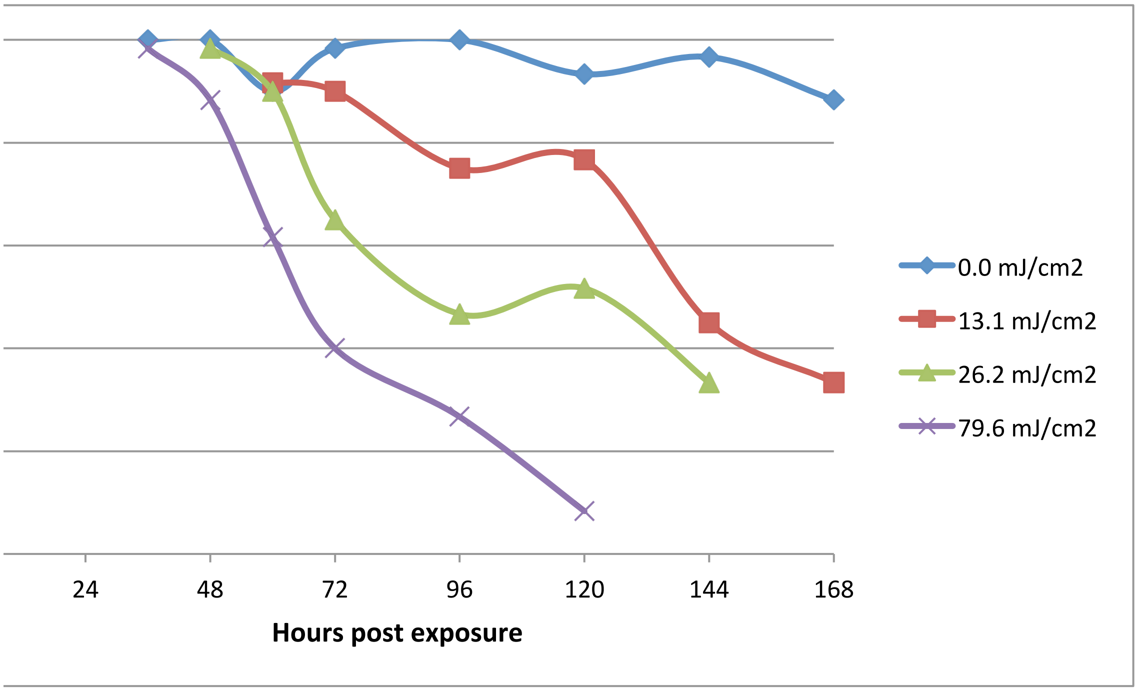

Supplement: S4 Datasheet — Collection data and exposure data from the fourth experiment. (PDF) [file pone.0133039.s004.pdf]
