# Supplementary figures and images for: The Effect of UV-C Exposure on Larval Survival of the Dreissenid Quagga Mussel
Source: PLoS One. 2015 Jul 17;10(7):e0133039. doi: 10.1371/journal.pone.0133039 (PMC4505903; doi:10.1371/journal.pone.0133039)

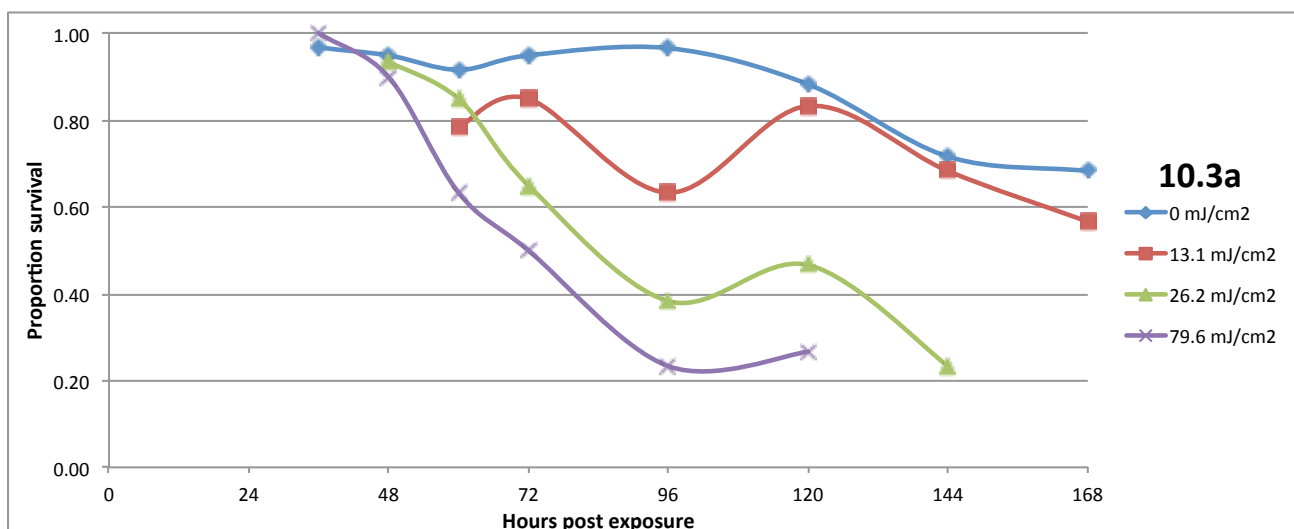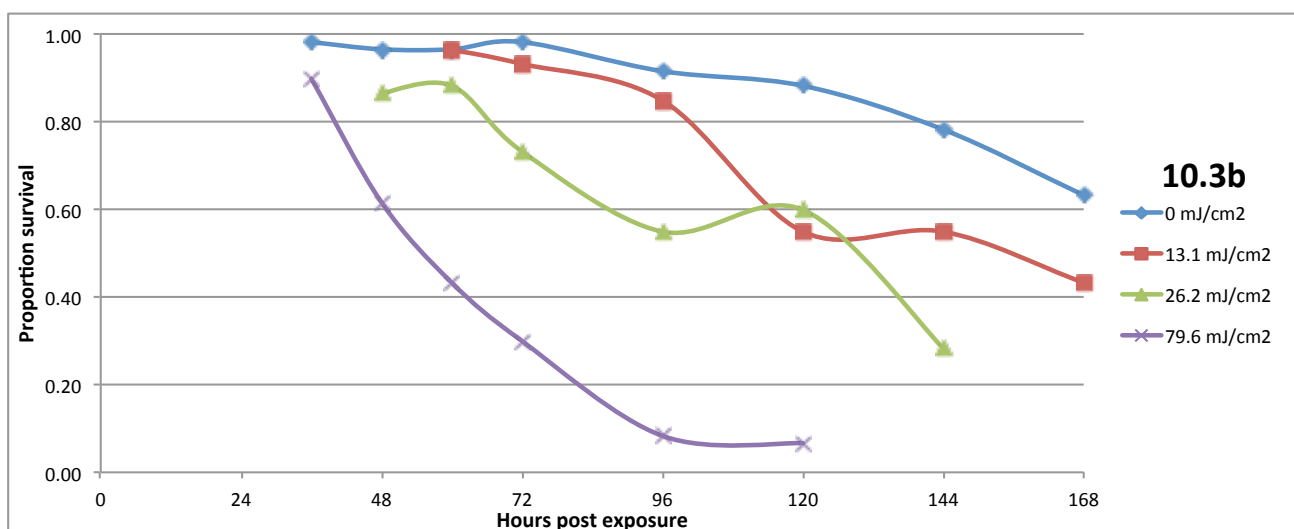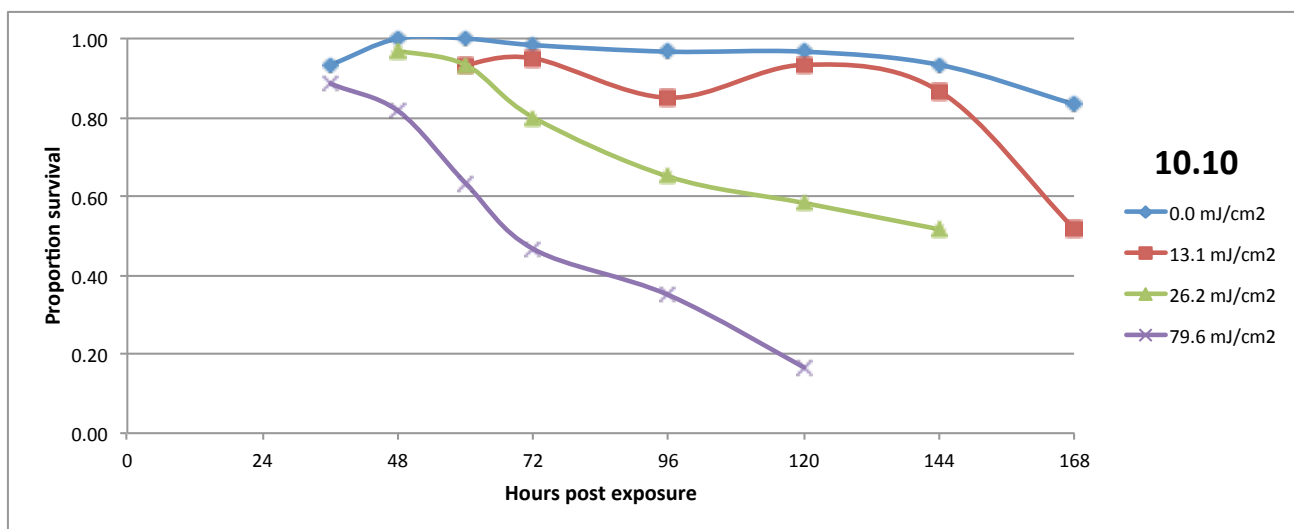

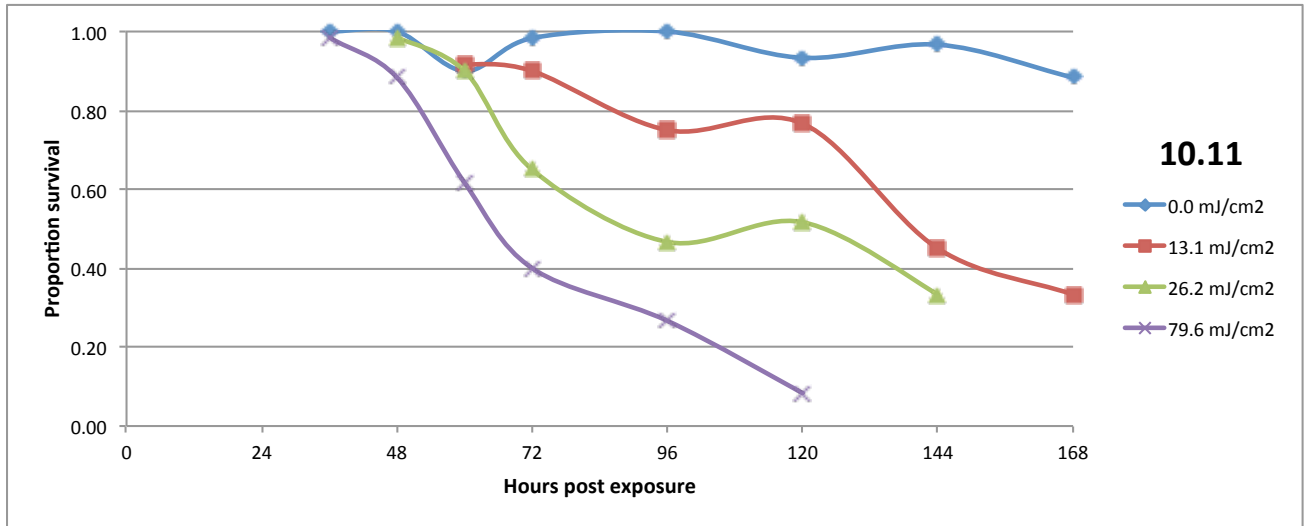

Supplement: S1 Table — Percent survival results from each individual experiment (one through four). (PDF) [file pone.0133039.s005.pdf]
